# The Role of Stabilizing Copolymer in Determining the Physicochemical Properties of Conjugated Polymer Nanoparticles and Their Nanomedical Applications

**DOI:** 10.3390/nano13091543

**Published:** 2023-05-04

**Authors:** Miao Zhao, Anton Uzunoff, Mark Green, Aliaksandra Rakovich

**Affiliations:** Physics Department, King’s College London, London WC2R 2LS, UK

**Keywords:** conjugated polymer nanoparticles, copolymer, stabilizing shell, physicochemical properties, nanomedicine

## Abstract

Conjugated polymer nanoparticles (CPNs) are a promising class of nanomaterials for biomedical applications, such as bioimaging, gene and drug delivery/release, photodynamic therapy (PDT), photothermal therapy (PTT), and environmental sensing. Over the past decade, many reports have been published detailing their synthesis and their various potential applications, including some very comprehensive reviews of these topics. In contrast, there is a distinct lack of overview of the role the stabilizing copolymer shells have on the properties of CPNs. This review attempts to correct this oversight by scrutinizing reports detailing the synthesis and application of CPNs stabilized with some commonly-used copolymers, namely F127 (Pluronic poly(ethylene glycol)-*block*-poly(propylene glycol)-*block*-poly(ethylene glycol) diacrylate), PSMA (poly(styrene-co-maleic anhydride)), PLGA (poly(D, L-lactide-co-glycolide)) and PEG (polyethylene glycol) derivatives. The analysis of the reported physicochemical properties and biological applications of these CPNs provides insights into the advantages of each group of copolymers for specific applications and offers a set of guidance criteria for the selection of an appropriate copolymer when designing CPNs-based probes. Finally, the challenges and outlooks in the field are highlighted.

## 1. Introduction

Conjugated polymers (CPs) are organic macromolecules characterized by a backbone chain consisting of alternating single- and double-bonds. The overlapping *p*-orbitals of this bond arrangement allow a delocalization of π-electrons across the adjacently-aligned orbitals, giving rise to most of the useful optoelectronic properties of CPs. This delocalized π-electron system can absorb light, resulting in generation and subsequent transport of charge carriers. The light energy absorbed can be effectively converted to fluorescence, heat, and other energies, making CPs optically and electronically active materials [1].

Due to the primarily organic composition of conjugated polymers, however, nanoparticles of CPs (CPNs) are intrinsically hydrophobic. This is in stark contrast to requirements imposed by most biological applications, e.g., imaging or therapeutic agents, which demand them to have good water solubility [2]. Two main approaches have been followed for the fabrication of water-soluble nanoparticles of CPs. The first involves the addition of polar side groups onto the side-chains of hydrophobic conjugated polymers, forming water-soluble conjugated polymers (WSCPs). The process endues WSCPs with amphiphilic character, directly ensuring the solubility of the resulting nanoparticles in aqueous environments, but also provides easy routes for further linking to biological entities such as antibodies [3]. However, more often than not, the addition of side chains induced a deleterious effect on the polymer’s optical properties, resulting in emission quenching; in fact, this ’super quenching’ was used as the basis of a sensing technology [4,5]. Conjugated polymer nanoparticles, on the other hand, were specifically designed to keep the emitting core polymer away from the linking components. In practice this is achieved *via* an addition of a stabilizing copolymer shell of amphiphilic character, that insulates the CP core from the potential harmful effects of the harsh linking chemistries used on the shell polymer. As a result CPNs are used as standard imaging agents, where the presence of light emission is consistent with the presence of the particles and whichever biological function they have been designed to highlight. Whilst some WSCPs can be used in similar applications such as cell imaging, the use of the CPN shell polymeric layer to protect the emitting polymer is the key difference between WSCPs and CPNs, with the stable light emission from CPNs providing a distinct benefit [3,6].

When it comes to the development of CPNs, the most popular method for their production is the nanoprecipitation method. In this method, CPs and an amphiphilic polymeric encapsulation matrix are both dissolved in an organic solvent prior to addition into a water phase under stirring or sonication [7,8]. When injected into aqueous solutions in this manner, the amphiphilic polymers, form micelle-like shells with their hydrophilic parts flaring out into the aqueous solutions and the organic parts flaring into the inside of the shells. In the presence of the hydrophobic CPs, such shells can encapsulate the CPs cores, yielding clear and transparent nanoparticles (NPs) dispersions [9].

**Figure 1:**
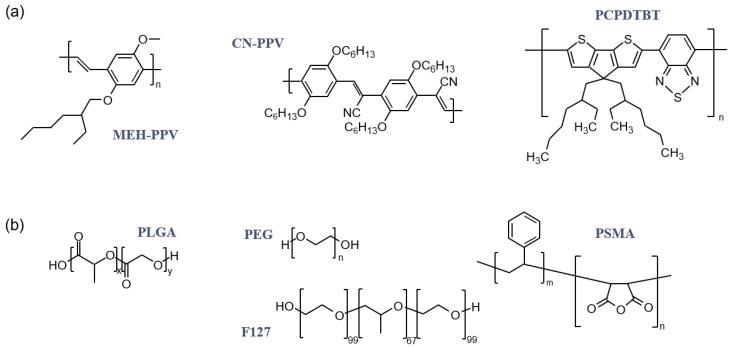
Chemical structures of common (**a**) conjugated polymers and (**b**) amphiphilic copolymers used in the fabrication of copolymer-stabilized CPNs.

Aqueous dispersions of different CPNs/amphiphilic polymer combinations can be produced via the nanoprecipitation method, and further surface functionalisations are possible with targeting ligands to enable specificity to CPNs probes’ bio-imaging function. Several review papers have been published summarising the various types of CPNs that have been previously produced, their ensuing physicochemical properties and biomedical applications [10,11,12,13,14]. It is not our intent to repeat this exercise; instead, the purpose of this review is to focus on those properties of the CPNs which are affected by the selection of the amphiphilic polymer used within the solubilising shells of the particles. We hope that in doing so, we provide a deeper insight into the reciprocities of the system that is a CPNs and offer more holistic CPNs design strategies. For accessibility, however, we limit our discussions to the most common amphiphilic polymers used for CPNs stabilisation: PSMA, F127, PLGA and PEG-containing polymers (see Figure 1 for their chemical structures) [15,16,17,18]. These copolymers have also been chosen since all of them are approved by the Food and Drug Administration (FDA) for pharmaceutical usage—the biocompatibility of the stabilizing shells is an important factor to consider, in view of the primary proposed application of CPNs as bioimaging agents.

PSMA is a copolymer typically composed of alternating styrene and maleic anhydride monomeric units. Styrene is hydrophobic, whilst maleic anhydride is hydrophilic; due to the opposing chemical nature of the two units, PSMA is solluble both in organic and aqueous solvents. This amphiphilic character can be exploited to encapsulate the inherently hydrophobic CPs and thus facilitate their solubility in aqueous environments [19]. The popularity of this copolymer in CPNs literature is at least partially due to its ready commercial availability in a broad range of molecular weights and maleic anhydride contents [20]. Another advantage is that, when forming CPs@PSMA CPNs, this copolymer can be a source of carboxyl functionalities for further surface modification requirements.

Pluronics are a commonly-used synthetic block copolymers that are composed of blocks of hydrophilic PEO (poly(oxyethylene)) and hydrophobic PPO (poly(oxypropylene)) polymers, arranged in a PEO_x_-PPO_y_-PEO_z_ triblock structure [21,22]. This triblock structure self-assembles into micelles in an aqueous environment in which PPO forms a hydrophobic core, and PEO forms a hydrophilic outer shell. Pluronics have different properties, depending on the relative values of x, y and z, and one of the most widely used Pluronics is the biocompatible Pluronic F127 (PEO_100_-PPO_65_-PEO_100_). Pluronic F127 has been used to prepare nanomicelles for drug and gene delivery. These applications have facilitated particle–cell interaction and enhanced cellular uptake efficiency [23,24].

PEG is a hydrophilic, often straight-chain polymer of ethylene oxide, whose general structure is HO–(CH_2_–CH_2_–O)_n_–H [25]. The amphiphilic nature of PEG varies depending on its molecular weight (MW)—low MW PEG polymers are more hydrophobic than high MW PEG polymers [26]. However, the PEG polymer has excellent biocompatibility, which makes it a popular choice for biological applications of PEG-coated CPNs [27]. PEG is the most popular polymer for drug delivery applications as it can inhibit the fast recognition by the immune system and leads to a reduced blood clearance of nanocarriers increasing blood circulation time [28]. Many different PEG-derived polymers are available, offering different end groups that could be used to functionalize the CPNs, e.g., further enhancing their cellular internalisation or achieving specific targeting. For instance, N_3_-PEG-NH_2_ could be modified with FA to develop a cellular probe [29], or the carboxyl end group of PS-PEG-COOH could be modified with streptavidin to achieve targeted imaging [30].

Due to the excellent biocompatibility and biodegradability, PLGA is one of the most frequently used biomaterials [31]. However, when used to prepare surface-modified polymeric nanoparticulate systems, it is most commonly combined with PEG into a hydrophilic polymer PEG(PLGA-PEG) [32]. PLGA-PEG combines all the advantages of PLGA and PEG, and as such it is considered to be one of the most promising systems for NPs formation and drug delivery applications [28,33,34,35,36,37,38]. Own its own, PLGA-PEG copolymer forms nano micelles with a relatively narrow size distribution of 10-100 nm; however, it can form core-shell architectures when loaded with a conjugated polymer, acting as a stabilizing shell instead [32]. One exciting opportunity that this copolymer offers is the ability to control the molecular weight of both the PLGA and the PEG components separately during the synthesis of PLGA-PEG, providing a pathway to fine-tune the encapsulation matrix of the CPNs in order to optimize its physicochemical properties [38].

In this review, we critically assess the use of each of the mentioned amphiphilic copolymers for the preparation of CPNs for bio-applications. The review is broadly divided into three sections, each dedicated to a different class of properties of CPNs that can be controlled *via* modification of their solubilizing shells. Unsurprisingly, we start with a basic discussion of CPNs solubility in aqueous solutions before moving on to various other properties affecting the possible applications of the CPNs and finally culminating in considering any biocompatibility implications. We then conclude with a summary of some general trends, observations, and omissions from the reviewed literature.

Note that, for clarity, we use the abbreviated names of polymers and chemicals throughout the text; the full chemical names and their corresponding abbreviations are given in alphabetical order in the Abbreviations section.

## 2. Aqueous Solubility and Colloidal Stability of CPNs

One of the key and critical requirements for any biomedical application is the chemical stability and water solubility of the probes/drugs upon which it relies [18,39]. As discussed in previous section, the use of CPs for such applications is limited by their poor water solubility - an issue that is usually overcome *via* an addition of a copolymer shell. It is important to note, however, that in this context the water solubility of the CPNs directly relates to their colloidal stability (please note that the terms “stability” and “colloidal stability” are used interchangeably in this text, to facilitate the ease of reading.), which are in turn determined by an interplay of various parameters such as the size of the particles, their zeta potential, as well as the pH, temperature and the ionic strength of the solution in which they are dispersed [40,41]. The addition of a copolymer shell, whilst generally expected to result in a slight increase of CPNs size, is proposed to improve the stability of the CPNs *via* an addition of charges to their surfaces. Despite this very common claim, however, very few reports directly compare stability indicators for shelled and unshelled (bare) CPNs and even fewer compare stabilities of CPNs with same core material but different copolymer shells. Nonetheless, some general conclusions can be made regarding the stability of CPNs in aqueous solutions by drawing on the common themes within the published literature, which are presented in this section.

### 2.1. Zeta Potential as Stability Indicator

The stability of a CPNs dispersion can be monitored using a number of different stability indicators, such as visual and/or spectroscopic inspection of solution clarity, investigations of changes to physicochemical properties of CPNs in response to environmental changes and over time, measurements of maximum concentrations attainable and so on. Amongst these, however, zeta potential—an important physical property that provides information about the charged interfaces’ electrical state—is the most commonly-used quantitative measure of the charge-induced colloidal stability of a NPs dispersion. As a general rule of thumb, zeta potential values of magnitudes greater than (30–40) mV are considered indicators of good electrostatic stability of NPs [42]. When it comes to literature pertaining to CPNs, however, this common indicator is still rarely reported (Table 1) and this severely hinders our ability to elucidate common trends and optimum practices. Nonetheless, some general observations can be made by examining the results presented in Table 1.

For example, a cursory examination of Table 1 leads to the conclusion that the use of PSMA and PEG-based copolymers seems to generally produce CPNs with increased surface charge density, when compared to cases where F127- or PLGA-PEG are used as stabilizing shells (Table 1). The zeta potential of PSMA-shelled CPNs tend to be very negative: the zeta potential of PBMC@PSMA CPNs was reported as −57.7 mV [15], and that of PFPtTFPP@PSMA CPNs as −33.4 mV [43]. For the F127-stabilized CPNs, on the other hand, the zeta potentials tend to be much less negative, as exemplified by PBTB@F127 CPNs [44] (−11 mV) and CN-PPV@F127+TMOS CPNs [45] (−12 mV), although in the latter case the CPNs were stabilized with TMOS-dopped F127. These observations were in line with those reported by Bourke et al., who performed the only known study of zeta potentials of CPNs of same core composition but different shell compositions [46]. In this work, the authors used both PSMA and F127 to encapsulate MEH-PPV CPs, concluding that the zeta potential of PSMA-stabilized CPNs were indeed larger in magnitude compared to F127-equivalent (−30 mV and −10.0 mV, respectively) [46]. CPNs stabilized by PLGA-PEG copolymer also tend to yield moderate surface charge densities, as illustrated by relatively neutral zeta potentials of F8BT@PEG_5K_-PLGA_55K_ CPNs (−4 mV to −10 mV) and CN-PPV@ PEG_5K_-PLGA_55K_ CPNs (−8 mV to −11 mV) [36]. As such, and based on zeta potential values alone, the reviewed literature seems to suggest that the use of PSMA and PEG-based copolymers should offer the best electrostatic stabilization of CPN dispersions. This reasoning, however, is somewhat misleading in that it does not take into account the encapsulation efficiency of the CPN with the copolymer shell (see next section for further discussions of this point).

Further examination of the zeta-potential reports in Table 1 shows that CPNs stabilized by PEG-based amphiphilic copolymers have received far better attention than other copolymers in this regard, with studies extending to investigations of zeta potential changes upon further functionalisations of CPNs. For example, Wang et al. found that upon modification of their PDPP-DBT@DSPE-PEG-Mal CPNs with the cell-penetrating peptide Tat, the zeta potential of the CPNs changed drastically from −34.4 mV ± 1.8 mV to 23.1 mV ± 1.7 mV [47]. The negative zeta potential of the unmodified DBT@DSPE-PEG-Mal CPNs was due to the negative charge of the maleimide (Mal) functional groups on the copolymer; conjugation of the positively-charged Tat peptide therefore increased the zeta potential of the CPNs. Using the same stabilizing copolymer, Wang et al. observed a similarly drastic change in the zeta potential of the CPNs when the DSPE-PEG-Mal copolymer was pre-modified with Tat before forming the CPNs: a value of −46.6 mV was reported for the the zeta potential of PTPEDC@DSPE-PEG-Mal CPNs, whilst the the zeta potential of the pre-modified PTPEDC-Tat@DSPE-PEG-Mal CPNs was 6.6 mV [48]. These reports show that care must be taken in further modifications of CPNs for targetting applications, so as to not de-stablize the CPN dispersion in the process.

For CPNs based on amphiphilic CPs, and therefore ones that possess an inherent surface charge, the zeta potential of the CPNs can be carefully controlled by electrostatic deposition of a copolymer of opposite charge to that of CP. For example, Liu et al. used a folate-conjugated cationic triblock copolymer PEI-PCL-PEG-FA to coat inherently negatively-charged PF-NPs through electrostatic interaction. By increasing the weight ratio of copolymer to PF CP, CPNs with zeta potentials ranging from −40 mV to +30 mV could be produced [49]. This ability to accurately adjust the electrical state of the CPN surface is of note, since it not only influences the aqueous stability of CPNs but also plays an essential role in determining their cellular uptake, cytotoxicity, and intracellular localisation. These themes are discussed in more detail in later sections; here, we merely wish to emphasise that CPNs’ zeta potential should form part of a comprehensive consideration when selecting a copolymer for a specific application.

**Table 1:** Physicochemical properties of bare and shelled CPNs.

Core Material	Shell Material ^†^	$\lambda_{\mathrm{ABS}}$ * (nm)	$\lambda_{\mathrm{PL}}$ * (nm)	PLQY (%)	D (nm)	ζ (mV)	Refs.
CN-PPV	*sln* (THF)	450	550	52	-	-	[45]
	*sln* (THF)	430	547	52	-	-	[36]
	F127 + TMOS	470	623	30	54 ± 3	−12	[45]
	PEG_5K_-PLGA_55K_	430	635	35	75	−8 to −11	[36]
CP1-4	-	750–816	-	-	-	-	[50]
	PSMA	-	-	-	49	-	[50]
DPP-TT	DSPE-mPEG_5K_	720	1100	-	90	-	[51]
EBKCP	*sln* (THF)	447	547	6	-	-	[52]
	PSMA	442	563	15	65	-	[52]
F8BT	*sln* (THF)	460	535	52–54	-	-	[36]
	-	460	540	22	29	−22 ± 6	[53]
	PEG	494	539	31	207	-	[54]
	PEG_5K_-PLGA_55K_	470	538	37 ± 1	105	−4 to −10	[36]
	PS-PEG-COOH	470	560	-	-	-	[55]
	PS-PEG-COOH	460	540	30	15	-	[30]
HCPE	PEG(N_3_-PEG-NH_2_)	355–361	409–415	30–40	10.8–13.5	-	[56]
MEH-PPV	*sln* (THF)	480	510	70	-	-	[46]
	*sln* (CHCl_3_)	498	560	27	-	-	[57]
	PSMA	-	540	25	60–140	−30	[46]
	F127	-	495	35	40–80	−10	[46]
	F127	512	590	15	61	0	[57]
	PLGA	-	590	-	271	−35	[58]
P2	PEG(N_3_-PEG-NH_2_)	375–505	640	1–12	130	-	[29]
PBIBDF-BT	-	811	-	-	-	-	[59]
	mPEG-b-PHEP	811	-	-	50	-	[59]
	PEG-PCL	782	-	-	156	-	[60]
PBMC	PSMA	417	558	2	44	−57.7	[15]
PBTB	-	635	-	-	-	-	[44]
	F127	330–500	420–653	-	192	−11	[44]
PCPDTBSe	-	764	-	-	150	−33.5	[61]
	F127	764	-	-	92	1.6	[61]
PCPDTBT	*sln* (THF)	690	760	67.7	-	-	[38]
	PEG_2K_-PLGA_4K_	670	850	2.3	-	-	[38]
	PEG_2K_-PLGA_15K_	650	850	7.5	-	-	[38]
	PEG_5K_-PLGA_55K_	640	850	1.1	-	-	[38]
	PEG_2K_-DPPE	650	850	7.5	-	-	[33]
PCPDTBT + PC70BM	PEG-b-PPG-b-PEG	650	840	-	54	-	[62]
pDA	DSPE-mPEG	654	1047	2	<6	-	[63]
PDPP3T	-	770	-	-	-	-	[64]
	F127	780	-	-	134.9	-	[64]
PDPP-DBT	DSPE-PEG-Mal	750	822	<0.1	100	−34.4 ± 1.8	[47]
	DSPE-PEG-Mal-Tat	750	822	-	-	23.1 ± 1.7	[47]
PFBD-N_3_	*sln* (C_6_H_5_CH_3_)	315,463	530	-	-	-	[65]
	*sln* (THF)	315,463	537	-	-	-	[65]
	*sln* (CHCl_3_)	315,463	549	-	-	-	[65]
	*sln* (CH_2_Cl_2_)	315,463	557	-	-	-	[65]
	PEG(N_3_-PEG-NH_2_)	320,468	585	11–17	-	-	[65]
PFBO	PS-PEG-COOH	550	603	-	-	-	[55]
PFBT	PLGA	-	560	-	243	−33.4	[58]
PF-DBT-COOtBut	-	370,545	-	1.1	-	−23.7	[66]
	F127	380,555	415,645	11.3	220	−10.68	[66]
PFO	-	385	419	-	-	-	[67]
	F127	380	439	63	105–142	-	[67]
	PSMA	375	430	-	10	-	[68]
PFODBT	-	535	705	2.8	31	−25 ± 5	[53]
	DPPC	535	695	2.5	35	−36 ± 7	[53]
PFP	PS-PEG-COOH	375	425	-	-	-	[55]
PFPE	-	340	375,393	-	-	-	[67]
	F127	345	400,419	76	100–137	-	[67]
PFPtTFPP	PSMA	375	651	3.3/9	21	−33.4	[43]
PFQ	PS-PEG-COOH	400	500	-	-	-	[55]
PFTBT5	PSMA	365	650	-	13	-	[68]
PTB7	-	675	780	0.5	140 ± 50	-	[69]
	F127	682	775	76	190 ± 60	-	[69]
	PSMA	380	765	76	150 ± 40	-	[69]
PtTFPP + PFO	Poly-L-lysine	440	650	-	110	45–53	[70]
PFVBT	-	365–502	612	-	-	-	[71]
	PSMA	502	598	-	120 ± 11	-	[72]
PFVBT + PIDTTTQ	DSPE-PEG_2K_-Mal	500	612	23 ± 1	34 ±0.9	-	[71]
PIDTTTQ	-	620–1100	-	-	-	-	[71]
Poly[9,9-bis(2-ethylhexyl)fluorene]	-	375	420	19	-	-	[49]
	PEI-PCL-PEG-FA	375	420	33	100	30	[49]
PTPEDC	DSPE-PEG-Mal	310	650	3 to 12	30	−44.2 to −46.6	[48]
	DSPE-PEG-Mal-Tat	310	650	-	-	−2.5 to −6.6	[48]
SP2	DSPE-mPEG_2K_	635–748	835	0.1–10	46	-	[73]

^†^ “-” corresponds to bare/unshelled CPNs dispersions in aqeous environments; “sln (X)” indicates solutions of CP in organic solvent X. * In all cases, the values reported are for the wavelength of the main absorption or emission maximum of the CPN dispersion. Where ranges of wavelengths are indicated, either multiple samples were reported with a range of peak wavelengths or same sample had multiple equal intensity peaks.

### 2.2. Improved Stability of Shelled CPNs

Almost every report detailing the fabrication and application of CPNs states that their solubility in aqueous environments is improved as a result of CPs encapsulation in a copolymer matrix. Yet, very few reports could be found that directly compare the colloidal stability of the bare and shelled systems, using any number of stability indicators. Still, the few reports that are available on this topic all support the stipulation. For example, in our own work, we found CPNs based on the hydrophobic PTB7 CPs to be unstable in aqueous environments, precipitating within 1–7 days post-fabrication, whereas F127- and PSMA-shelled PTB7 CPNs showed no signs of aggregation for time periods exceeding 6 months [69]. In addition, both of our shelled CPNs systems were stable against ultracentrifugation, allowing concentration of these samples to 420 μg.mL^−1^, whereas a much reduced maximum concentration of 90 μg.mL^−1^ could be obtained with bare PTB7 CPNs, without causing their irreversible aggregation. An interesting further observation made in our work was the change in CPNs hydrodynamic diameters over a 6 month period: all three samples saw a reduction in their average sizes, with F127-shelled CPNs showing the smallest reduction of the order of 1–2%, PSMA-shelled CPNs demonstrating a slightly larger reduction of 10% and bare PTB7 CPNs exhibiting a 50% reduction. This observation is most likely caused by CPs chains leaking from the CPNs core, similar to that observed by Kai et al. [58], and it appears that the larger F127 copolymer (MW of 12.6 kDa) provided better protection against this than the smaller PSMA copolymer (MW of 1.9 kDa), but either of the copolymers improved the long-term stability of the CPNs.

MacNeill et al. likewise observed an improvement in the stability of their CPNs based on PCPDTBSe CPs, with the average stability of the samples in water, PBS or cell culture media improving from ≈1 week to >2 months upon addition of a F127 copolymer shell [61]. This report is interesting because the improvement in stability that the authors observe goes against the trend observed in their zeta potential (−33.5 mV for bare CPNs to 1.6 mV for F127-shelled CPNs, Table 1). This results seem to indicate that the electrostatic stabilization provided by the amphiphilic character of the PCPDTBSe CPs itself was not sufficient to achieve good colloidal stability of bare CPNs; however, encapsulation in F127 micelle, despite causing a reduction in the zeta potential of CPNs, could provide sufficient steric stabilization to produce colloidally-stable CPNs dispersions.

**Figure 2:**
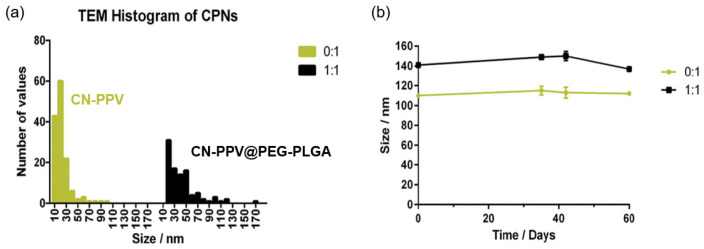
(**a**) TEM particle size distribution of CN-PPV (0:1) and CN-PPVPEG-PLGA (1:1) CPNs. (**b**) Colloidal stability of CN-PPV (0:1) and CN-PPVPEG-PLGA (1:1) systems stored in the dark at $37{\;}^{{}^{\circ}}$C over 60 days, as measured by DLS. Adapted from reference [37] with permission from the Royal Society of Chemistry.

Kemal et al. also used an amphiphilic core CPs in their work (CN-PPV) and encapsulated it in a somewhat less amphiphilic diblock copolymer PEG_5K_-PLGA_55K_, resulting in a decreased magnitude of the zeta potential for the shelled CPNs when compared to bare ones (−77.1±0.6 mV for CN-PPV and −43 mV to −55 mV for the CN-PPV@PEG-PLGA CPNs) [37]. They report that both systems—the bare CN-PPV CPNs *and* CN-PPV@PLGA-PEG CPNs—were colloidally stable for over 60 days when kept under incubation conditions ($37{\;}^{{}^{\circ}}$C). The authors based their conclusion on measurements of CPNs sizes, which do indeed stay stable over the specified period, as shown in Figure 2. The results seem to suggest that the core polymer itself is sufficiently amphiphilic to form stable colloidal dispersions and the addition of small amounts of PEG-PLGA (with less amphiphilic characteristics) does not negatively impact the colloidal stability of the CPNs. Interestingly, however, the authors also report that both of these CPNs systems aggregated irreversibly upon centrifugation at relatively low speeds. It is only by increasing the mass ratio of the stabilizing copolymer to CPs polymer, and thereby further decreasing the zeta potential of their particles (−29.2±0.4 mV), that CPNs with sufficient stability to withstand the centrifugation process could be produced. Likely, this is due to an improved encapsulation of CN-PPV core for higher PEG-PLGA:CN-PPV mass ratios, preventing unravelling of the CPNs under the stresses imposed by the centrifugation process, compared to lack of such ability for lower mass ratios due to an incomplete encapsulation of the core.

In view of these reports, a tentative hypothesis can be made that efficient encapsulation is critical to both the short-term and long-term stability of the CPNs, regardless of whether the CPs at the core of CPNs has amphiphilic or hydrophobic character—the copolymer shell *can* act to electrostatically stabilize a CPN dispersion in the case of the latter, but it *always* acts as a prohibitor to CPN unravelling and/or CPs leakage. Furthermore, these reports provide first indicators that copolymers of larger molecular weight might perform better as encapsulating agents. This hypothesis is somewhat supported by the work of Kai et al. who evaluated the encapsulation efficiency and CPs leakage for four different CPs encapsulated in the same PEG-PLGA matrix [58]. They observed slight differences in the encapsulation efficiency of the four CPs and an opposite trend in the CPs leakage data, i.e., amount of CPs leaked from the CPNs over a period of 5 days was higher for CPNs with poorer encapsulation efficiency (data shown in Table 2).

As such, and despite their few numbers, investigations that compare stabilities of bare and shelled CPNs elucidate complex inter-relations between the different parameters that determine their colloidal stability in aqueous solutions and provide guidance in the design of colloidally-stable CPN dispersions.

### 2.3. Stability of CPNs in Different Environments

It is crucial to investigate the stability of CPNs in physiological environments because CPNs can interact with biological systems in various ways, including through cellular uptake, adsorption to cell membranes, and interaction with biomolecules such as proteins. These interactions can affect the behavior and effectiveness of the CPNs in biomedical applications. For example, if CPNs are not stable in physiological environments, they may aggregate and/or change their shape or size, both of which can affect their efficacy and safety. Moreover, unstable CPNs may induce unwanted immune responses or toxicity, which can limit their clinical use. Therefore, understanding the stability of CPNs in physiological environments is essential for the rational design of safe and effective NPs-based therapeutics and diagnostics. It can also help in developing appropriate strategies for the synthesis, surface modification, and functionalization of CPNs to improve their stability and performance in biological systems. Three key attributes can be identified when comparing physiological environments to those of as-produced aqueous dispersions of CPNs: the temperature, the pH and the presence of biomolecules such as proteins in the environment. Here, we examine the effect of these attributes on the stability of CPNs.

Regarding the effects of the temperature, the stability of CPNs at $37{\;}^{{}^{\circ}}$C is of particular interest as this reflects the human body’s temperature. Currently, most studies of CPNs stability at $37{\;}^{{}^{\circ}}$C use PEG as the copolymer, which is likely due to its general popularity as a polymer material that is known for its excellent biocompatibility with wide biomedical applicability, where it contributes towards the long-term and synergistic effects of the drugs which contain it. Pertaining to its use as a CPN stabilizer and the resulting temperature-dependent stability of the CPNs, Yuan et al. investigated the stability of PFVBT coated with N_3_-PEG-NH_2_, and found that the fluorescence of PFVBT@N_3_-PEG-NH_2_ and size remained unchanged even after incubation in water, PBS, and DMEM cell medium for 7 days at $37{\;}^{{}^{\circ}}$C [72]. Similarly, Feng et al. found that the fluorescence intensity of (PFVBT+PIDTTTQ)@DSPE-PEG2000-Mal remain unchanged after incubation for 10 days in PBS buffer at $37{\;}^{{}^{\circ}}$C as well as after 5 days incubation in serum containing cell culture medium [71]. There are a smaller number of studies on CPNs stability at $37{\;}^{{}^{\circ}}$C using PSMA as coating polymer. Liu et al. found the fluorescence intensities and the zeta potential of CPNs using PSMA as copolymer remained nearly unchanged after incubation in PBS at $37{\;}^{{}^{\circ}}$C for up to 10 days [69]. These results seem to indicate that CPNs can remain stable under physiological temperatures regardless of the nature of the copolymer shell, at least over short periods of times such as those that would be involved in the use of CPNs as e.g., bioimaging agents or therapeutic probes. However, these are is no consistency nor completeness in what individual pieces of research report as the stability indicator(s). This is crucial since, and as discussed in previous sections, lack of changes in one stability indicator is not necessarily sufficient to draw definite conclusions as to overall stability of a CPN dispersion.

Many biological environments are characterized by different pH conditions and any probe that is considered for bio-applications must be stable within the pH range that it will encounter from the point of injection or application and to the tissue that it is targetting. In biomedical applications of CPNs, they key environments to consider are those of normal and tumorous tissues, with the latter generally being somewhat more acidic [74,75]. As is typical of any colloidal dispersion, CPN stability can indeed be affected by changes of the environmental pH. Yet, this is not a topic that has been investigated in the literature, with the exception of a single report on PTB7 CPNs [69], which happens to be our own previously-published work. In this work, we found that the hydrodynamic sizes of F127-shelled PTB7 CPNs remained consistent throughout the pH range of 4 to 10, which was consistent with the minimal changes observed in the absorption spectra for these samples. This did not translate to PSMA-solubilized PTB7 CPNs, however, for which notable changes were observed both in the DLS data and in the absorption spectra. The above results indicate that F127 provides better stability against pH changes when compared to PSMA; however, given the extremely-limited availability of relevant studies, the underlying mechanism behind this observation remains unclear.

On the topic of stability of CPNs in the presence of biomolecules, PEG-stabilized CPNs have received the most attention. However, amongst the three groups of copolymers under consideration in this review, F127 has shown the best long-term stability in such physiological environments. For example, PCPDTBSe@F127 CPNs showed stability of longer than 2 months in water, PBS, and Hybricare or Roswell Park Memorial Institute 1640 medium supplemented with 1% L-glutamine, 1% penicillin/streptomycin, and 10% fetal bovine serum [61]. In contrast, the longest stability reported for PEG- and PSMA-stabilized CPNs in similar environments was seven and ten days, respectively [72,76]. The results above indicate that although there are fewer studies on the stability of F127 as copolymer compared to PEG, it provides excellent long-term stability in the presence in biologically-relevant environments. These findings suggest that F127 has significant potential for applications in this field, and further research in this area is warranted.

Overall, this section highlights the need for further research into the stability and response of CPNs of different compositions to changes in their local temperatures, pH values, and solution environments. Understanding the stability of CPNs in these conditions will contribute to their effective application in various fields, including drug delivery, imaging, and theranostics.

### 2.4. Long Term Stability

Above considerations of stability point towards the possibility that copolymer selection is crucial in guaranteeing long-term stability of CPNs and preventing any degradation in their functionalities, such as loss of quantum yield (QY) or reactive oxygen species (ROS) production, or changes to their surface chemistry. Copolymers that result in no measurable changes of sizes of CPNs, their surface potential, absorption/emission, and other properties over a long period of time should be preferred since they provide a viable option for the long-term storage of CPN-based pharameceuticals and bioimaging agents. As such, there is a great need for in-depth studies of long-term stability of CPNs at storage temperatures (room temperature or $4{\;}^{{}^{\circ}}$C are most convinient). Yet, only a hand-full of reports exist quoting any stability indicators of CPNs over time periods longer than month. Of these, five reports utilize visual inspection of dispersion clarity and colour as the only stability indicators; as discussed previously, such inspections are not deterministic, as they do not take into account the possible unravelling CPNs and leakage of CPs into the solution. Nonetheless, visual inspections are still useful and should be quoted, as is illustrated by the report of Bourke et al. who observed that CN-PPV@F127+TMOS samples kept at $4{\;}^{{}^{\circ}}$C remained clear for periods of more than three months, whereas those stored at room temperature showed clear signs of aggregation after 2 months [45].

Second to visual inspection, CPN size variability and/or changes in absorption and emission spectra have also been used as stability indicators. The resounding conclusion seem to be good colloidal stability during the storage for all three copolymers, although very few exist for PSMA- and F127-shelled CPNs. For PEG-based copolymers, Lu et al. reported on DPP-TT CPNs shelled with a DSPE-mPEG_5K_ copolymer - they observed negligible DLS size changes after 30 days of storage, as well as insignificant DLS changes after both irradiation and temperature modulation, indicating impressive stability of their CPNs [51]. Wang et al. studied PTPEDC CPNs shelled with a similar copolymer, DSPE-PEG-Mal, and reported that 3 months of storage resulted in almost no change in DLS measurements (30±1 nm to 33±1 nm) and no shifts in the positions of PL and absorption peaks [48]. Pu et al. looked at the use of SP2 CPNs shelled with the DSPE-mPEG_2K_ copolymer for photoacoustic imaging; again, they reported no significant DLS changes over a storage period of three months [73].

Only a single report exists where the long-term stability of PSMA- and/or F127-shelled CPNs was investigated; this is our own work on PTB7 CPNs, which used DLS size and spectral investigations to conclude that F127-shelled CPNs remained stable for more than 6 months, whereas PSMA-shelled CPNs showed some signs of CPN unravelling and CP leakage, as previously discussed in Section 2.2 [69]. The lack of information for these two copolymers is worrying in view of their popularity in CPN-based systems. A more concerning commonality amongst the reports, however, is the lack of reporting of zeta-potentials of CPNs stored for long periods of time, on account of it playing a major role in almost every facet of CPNs’ proposed medical applications. As it stands, no further conclusions can be made regarding the stabilities of CPNs shelled with different copolymer. To develop a complete and comprehensive understanding in this regard, further investigations of CPN stabilities are necessary, with a bare minimum reporting set consisting of CPN sizes, optical properties and their zeta potentials, all monitored over time periods exceeding a month.

## 3. Optical Properties of CPNs

It is generally accepted that CPNs’ optical properties originate from the optoelectronic response of the CP materials that form their core [77]. Surprisingly, however, some changes in the optical properties of CPNs has been observed when they were shelled with amphiphilic copolymers. Here, a review of these reports, together with some key observations, are described. Much of the key information using which we draw our conclusions is summarized in Table 1.

### 3.1. Shifts in Optical Spectra

The optical spectra (absorbance and photoluminescence) of CPs does often shift after being encapsulated in copolymers when compared to spectra of CPs in the organic solvent; the direction and degree to which this happens, however varies case-by-case. This is reflective of the origin of the optical properties of conjugated polymers and their dependency on the chain packing and/or disorder in the nanoparticulate system.

**Figure 3:**
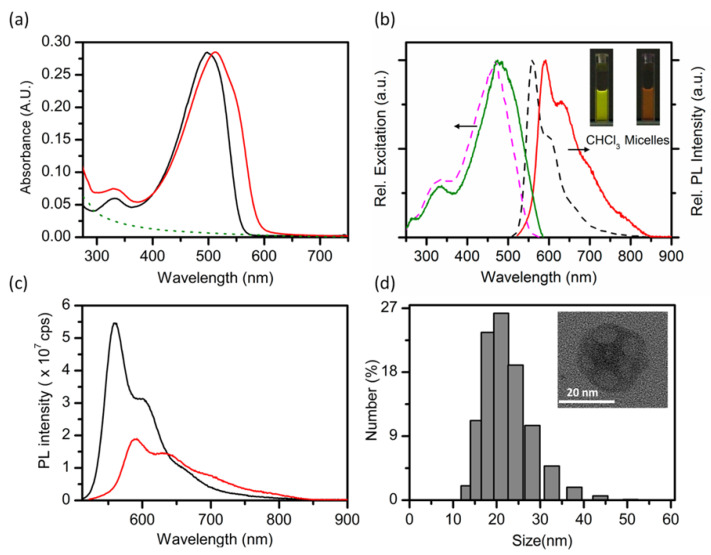
Comparison of properties of MEH-PPV in an organic solvent and as a CPNs in aqueous solution. (**a**) Absorption spectra of a MEH-PPV solution in chloroform (black line) and the corresponding conjugated polymer nanomicelles prepared in water (red line). The dashed green line is an absorption spectrum of empty F-127 micelles. (**b**) Intensity-normalised excitation and emission spectra of a MEH-PPV solution (dashed lines) and MEH-PPV micelles (solid lines). Insets: photographs of MEH-PPV emission from a chloroform solution and a micellar suspension under black light illumination. (**c**) Emission spectra of MEH-PPV in chloroform solution (black line) and aqueous nanomicellar suspension (red line). (**d**) Number-average hydrodynamic size distribution of MEH-PPV micelles. Inset: transmission electron micrographs of a typical MEH-PPV micelle. Reprinted with permission from [57]. Copyright 2016 American Chemical Society.

Interestingly, for F127-stabilized CPs, more often than not, the resulting CPs show a red-shift in their optical spectra (Table 1). For example, as can be seen in Figure 3a, the absorbance peak of MEH-PPV is 498 nm in a chloroform solution, but it red-shifts to 512 nm when co-precipitated into water in the presence of F127 to form F127-shelled CPNs. This is coincident with a red-shift in the emission spectrum of MEH-PPV in the two environments. The authors speculate that the observed red-shifts in spectra of CP upon encapsulation in F127 micelles are due to the formation of a fraction of red-shifted aggregates with an energy disorder, which are themselves a result of increased interchain interactions in the CP core [57]. Despite the common occurrence of red-shifts in the optical spectra of CPs stabilized with F127, there have also been a few instances in which blue shifts or no shifts have been observed (Table 1) for such particles. For instance, Ye et al. observed a slight blue-shift in the absorbance of PFO@F127 aqueous solution compared to PFO in tetradyrifuran (THF) [9]. Similarly, Bourke et al. reported a blue shift in both absorption and emission spectra of MEH-PPV CPs upon encapsulation with F127 compared to those encapsulated by PSMA [46]. However, when PFPE was encapsulated with F127, no significant changes in absorbance were observed when compared to PFPE in THF [9].

**Figure 4:**
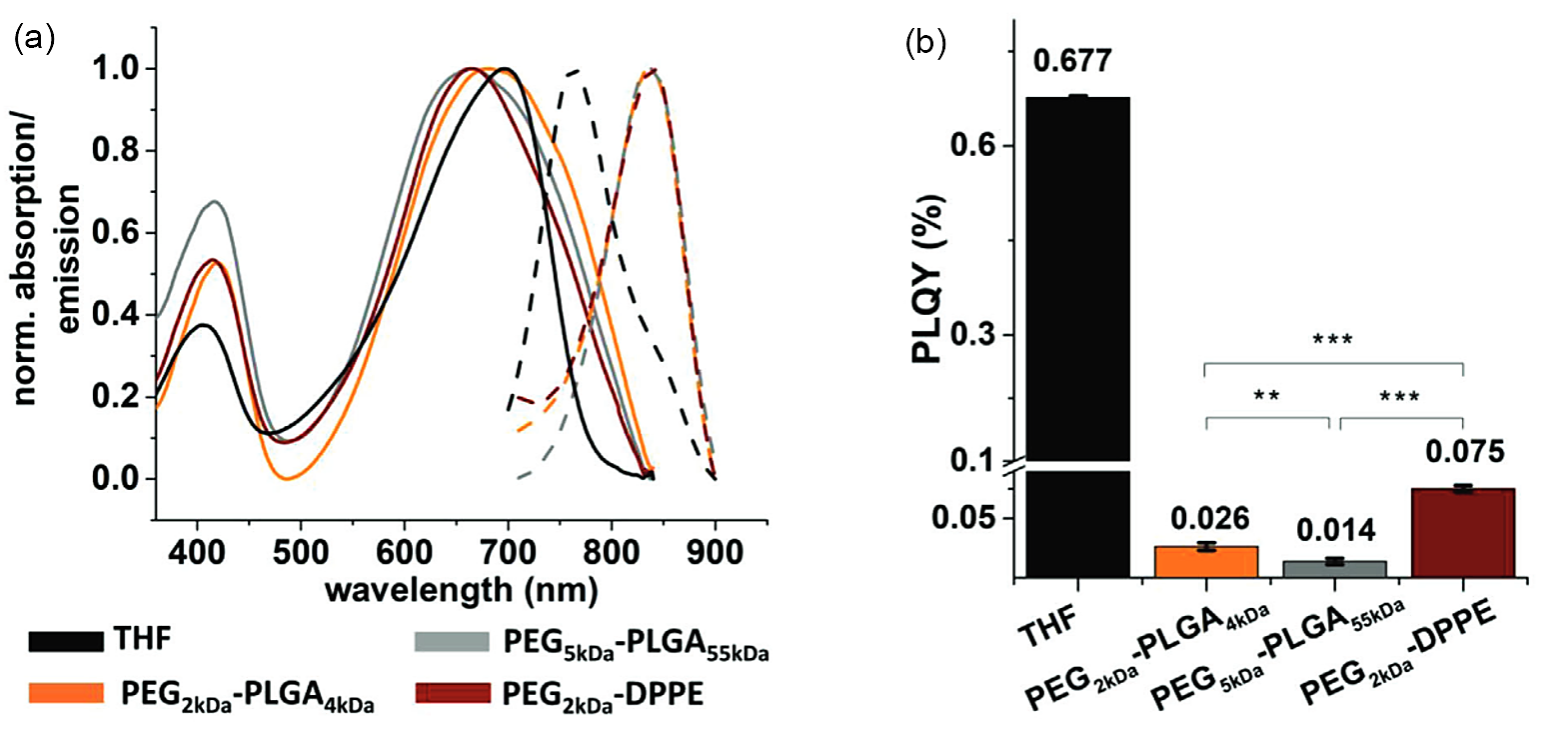
Comparison of optical properties of PCPDTBT CP in solution and as core of a CPN. (**a**) Normalized absorption (solid lines) and emission spectra (dashed lines) of PCPDTBT in THF and 5% PCPDTBT CPN dispersions. (**b**) PLQY of PCPDTBT in THF and 5% dispersions of PCPDTBT CPNs. PLQY values were directly measured in an integrating sphere at a fluorophore concentration of 1.7 μg.mL^−1^. All values represent the mean ± standard deviation of n=3 independently produced batches per formulation. *p*-Values were calculated using one-way ANOVA with Tukey’s post hoc test, * p<0.05, ** p<0.01, *** p<0.001. Adapted from [33], with permission from John Wiley and Sons.

An interesting alternative observation of spectral changes upon formation of the CPNs is the opposing shift of the absorption and emission peaks, typically characterized by a blue-shifted absorption features and red-shifted emission peaks. This was, for example, observed by Neumann et al., as shown in Figure 4, when PCPDTBT was encapsulated in PLGA-PEG [33]. Compared to PCPDTBT in THF, PCPDTBT@ PLGA-PEG NPs shows 20–50 nm blue shift of absorption peak, which the authors attribute to the reduction in the length of π-conjugated domains through bending, torsion and kinking of the PCPDTBT polymer chains. The CPNs formulations also show an 80 nm red shift in the emission peak compared to the THF solution, which is also caused by the spatial changes in the polymer structure, causing an increased interchain interactions and the aggregate formation.

### 3.2. Photoluminescence Quantum Yield Changes

When CPs are precipitated into nanoparticulate form, their photoluminescence quantum yield (PLQY) typically decreases strongly when compared to emission from fully solvated polymer chains in organic solvents. For example, Neumann et al. reported a 10–50-fold decrease of the PLQY of PCPDTBT@PLGA-PEG CPNs when compared to PCPDTBT dissolved in THF [33]. The same authors also report that the choice of stabilizing copolymer can influence the degree to which this happens. For example, a three- to five- fold improvement in the PLQY of their CPNs could be achieved by replacing the PLGA-PEG copolymer with PEG_2K_-DPPE during the nanoprecipitation process (see Figure 4). That being said, the choice of the solubilizing copolymer is not the only factor to determine the optical performance of the CPNs - variations in the fabrication procedure have also shown to be a factor; Abelha et al. found that even the same CPs encapsulated within the same copolymer matrix exhibit different optical properties when prepared through different manufacturing procedures [36]. Specifically, they compared the use of a microfluidic technique and a conventional solvent displacement method to manufacture CN-PPV and F8BT CPNs stabilized by the PEG_5K_–PLGA_55K_ copolymers. Their key observations were that the solvent displacement method produced smaller NPs (75–200 nm) than the microfluidic method (140–260 nm), with a corresponding trend in the PLQY of the prepared CPNs (the microfluidic technique produces CPNs of consistently higher PLQYs) [36]. Most likely, this is a direct relation, with the smaller PL QYs of smaller NPs being a result of increased contortion of the polymer chains, leading to increased interchain interactions and/or aggregate formation due to tighter packing of chains.

When it comes to optimization of fluorescence properties via an appropriate selection of stabilizing copolymer, PSMA deserves particular attention. In general, its addition to the NPs composition at the right copolymer-to-conjugated polymer ratio will result in an improvement of the PLQY of the CPN, when compared to bare CPNs prepared in the absence of any stabilizing molecule [69,76]. However, the use of other copolymers, such as F127, has been shown to result in better improvements of the emission properties, as was shown for MEH-PPV and PTB7 CPNs [46,69]. In their work, Liu et al. stipulated that the hydrophobic ends of PSMA are able to interpenetrate the CPs chains, reducing the contact between individual chains and, in their case, reducing the energy transfer between the donor and acceptor units [76]. It is likely that similar effect is responsible for reduced fluorescence from CPNs characterized by H-aggregate emissions (which partially rely on interchain interactions) and that use of other copolymers, such as F127, mitigates this issue in such cases.

### 3.3. Photostability Changes

Excellent photostability of CPNs suspensions in an aqueous medium is crucial for clinical applications involving continuous irradiation of probes over extended periods of time, which include imaging of tissues and phototherapy. Yet, few reports can be found of any investigations of the photostability of CPNs suspensions in the literature. The few examples that do exist, however, are promising in that most report excellent photostability of CPNs when stabilized by any copolymers. For example, upon a continuous laser excitation at 458 nm (5 mW) over 10 min period, the fluorescence intensity of PSMA-encapsulated EBKCP CPNs decreased by only 10% [52]. Li et al. investigated the in vitro photostability of PFV@PLGA CPNs by continuous laser excitation at 405 nm for 20 min during cell imaging, and detected no observable changes in the levels of the bright green fluorescence from the CPNs [58]. A similar observation was also made by Neumann et al. for PEG-PLGA-stabilized PCPDTBT CPNs when used for imaging of a phantom mouse, who report a less than 20% decrease in the signal-to-noise ratio after total irradiation time of approximately 100 h [33].

The limited number of detailed investigations of the photostability of CPNs stabilized by different copolymers make it difficult to draw any conclusions as to any dependencies that may exist between the two. The only exception to this is, perhaps, our own work, where the photostability of bare, PSMA- and F127- stabilized CPNs was directly compared [69]. In this work, we showed that encapsulation of CPs in copolymers did improve the photostability of the particles under prolonged excitation, but the degree to which this happened was similar for the two copolymers. However, in further stability studies desribed within the same work, the PSMA-encapsulated CPNs were shown to be more sensitive to changes in the pH of the environment and the presence of serum when compared to F127-encapsulated PTB7 CPNs (Supplementary Information, reference [69]). Therefore, and based solely on the photostability arguments, F127 and PEG-based copolymers would seem to be the better candidates for bioimaging applications; however, and as we will discuss in the next few sections, these arguments are insufficient to make such claims.

## 4. Cell Targeting and Uptake

NPs are known to undergo increased cellular uptake and accumulation in tumour tissues, as compared to normal tissues, due to the enhanced permeability and retention (EPR) effect [78]. However, the EPR effect is heterogeneous amongst tumour types and such the tumour-dependent characteristics limit the usefulness of the EPR effects in bio-applications [79]. The use of CPNs functionalized with *targeting* entities often results in better imaging contrast than relying on *passive* CPNs accumulation in the tumor via the EPR effect [80]. The reason for this is that cell adhesion and uptake of the NPs is strongly dependent on cell type, copolymer on the CPNs surface and the interaction of the two. A CPNs stabilized by a specific copolymer may have a strong affinity to some cells, have poor affinity to others or non-specifically adhere to almost any type of cells. In cases where CPNs naturally have poor affinity to specific cell types, additional functionalisation can improve cell targeting and uptake; however, this can only be achieved if functional groups on the surface of the CPNs are available for further functionalization [81]. Incorporating different polymeric matrices during CPNs preparation is an efficient method to introduce surface functional groups to facilitate their bio-applications [79], and this property is common amongst all four types of copolymers under review here.

According to the articles reviewed, PSMA and PEG-based copolymers are excellent shell materials for use in CPN-based probes designed for *general* bioimaging applications. CPNs encapsulated in both of these materials have a tendency to adhere naturally to most cell types; however, the shells also provide easy chemical routes for more specific targeting via click chemistry or carbodiimide reactions [47,68,71,82]. On the other hand, encapsulation with F127 tends to result in a much more selective cellular affinity, with strongly inhibited non-specific cellular uptake of CPNs [69]. As such, further modification of F127-shelled CPNs has the potential to produce highly specific cellular probes for targeting cancerours tissues. In fact, the only reports of cell adhesion or internalization for F127-solubilized CPNs without any further modification have been for the HeLa cell line. For example, Kim et al. used PBTB@F127 CPNs for imaging of HeLa cells, and concluded that their CPNs diffused into the cytoplasm of the cells [44]. Bourke et al. utilized MEH-PPV CPNs, encapsulated in silica-shell cross-linked F127 micelles, and observed bright fluorescence of these CPNs within HeLa cells [46].

When considering cell targeting and uptake for CPNs, the use of copolymer PSMA as solubilizing shells presents an excellent opportunity since, during the formation of CPNs in water, the maleic anhydride units of the PSMA are hydrolysed, generating carboxyl groups. Using standard carbodiimide chemistry, these readily available carboxyl groups can then be reacted with the amine groups on antibodies, affording desired CPNs-antibody conjugates. For example, this strategy was successfully implemented by Feng et al. to achieve targeting of specific cell lines, depending on the antibodies they have attached (anti-EpCAM or anti-ErbB2) and the level of expression of corresponding antigens by the tested cell lines (SK-BR3, MCF-7 and HeLa) [82]. Via the same route, Chen et al. functionalized PSMA-solubilized CPNs of PFO and PFTBT5 CPs with streptavidin, for use in super-resolution fluorescence imaging. They found that the resulting CPNs could specially target specific subcellular structures with high labelling density in BS-C-1 cells, including mitochondrial outer membranes, cytoskeleton microtubule filaments, and clathrin-coated vesicles [68].

Wu et al. took an alternative approach to exploiting the natural hydrolyzation of the PSMA in water—they employed the carbodiimide chemistry to first functionalize their CPNs with azido and alkyne groups via a reaction with corresponding variants of amino-terminated PEG [20]. They then proceeded to use the copper (I)-catalysed click chemistry reaction to attach a small amount of fluorophores to the CPNs, whose fluorescence could then be used for bioimaging. The functionalization of CPNs with azido and alkyne functional groups is significant, since they are considered to be bioorthogonal, i.e., they have no inherent interactions with any native biological groups, providing enhanced selectivity in cell labeling applications.

In stark contrast to these works, lack of any modification of the carboxyl group of the PSMA has been associated with generally non-specific binding to cell membranes [76], or endocytosis-driven accumulation of CPNs in lysosomes [52].

The PEG-derived copolymers also offer several routes to achieve enhanced cellular internalisation or specific targeting. Firstly, many PEG derivatives are available, providing a facile route to ensure specificity of the CPNs even at the fabrication stage. However, a more common approach is to employ common variants with easily reactable functional groups, and impose the specificity following a further conjugation step with a targeting element. For instance, Wang et al. developed CPNs of PDPP-DBT encapsulated with DSPE-PEG-Mal copolymer, and the maleimide groups terminated at the DSPE-PEG-Mal chain ends were then used to conjugate with a cell-penetrating peptide (Tat) [47]. The resulting CPNs were NIR-active, and possessed photothermal properties, which meant that they could effectively coat the surface of HeLa cells and generate localized heat to trigger target gene expression. Wang et al. also used the same copolymer DSPE-PEG-Mal modified with Tat to encapsulate PTPEDC to achieve two-photon excited PDT therapy in Hela cell [48]. The maleimide groups terminated at the DSPE-PEG-Mal chain ends have also been decorated with anti-HER2 affibody by Feng et al., who synthesized CPNs based on PFVBT and PIDTTQ, and DSPE-PEG_2K_-Mal and achieved superior selectivity towards tumour cells with HER2 overexpression [71].

Other common PEG derivatives employed for the purposes of CPN solubilization include PS-PEG-COOH. Similarly to DSPE-PEG-Mal, PS-PEG-COOH can also be modified with Tat through a covalent link. Zhou et al. developed multiple wavelength emission CPNs with different fluorene derivatives as a core and PS-PEG-COOH as an encapsulation matrix. To perform the biological applications, CPNs were further modified with Tat and successfully employed in fluorescence imaging of A549 cells [55]. Wu et al. demonstrated bioconjugation of ultrabright Pdots for specific cellular targeting [30]. They encapsulated PFBT in the PS-PEG-COOH matrix, and then further functionalized their Pdots with streptavidin via the EDC coupling reaction. The developed Pdots and the anti-EpCAM primary antibody were then sequentially incubated in MCF-7 cultures, to achieve effective and specific cell targeting.

An interesting observation regarding the use of the PEG-derived copolymers is that the length of the PEG chain seems to be a factor that can influence the cell uptake efficiency. Liu et al. compared the uptake of PFBD-PEG_0.6K_-COOH NPs and PFBD-PEG_2K_-COOH NPs in MCF-7 cell cultures [65]. They determined that PFBD-PEG_2K_-COOH NPs (the larger of the two) were less efficiently taken up by cells, which significantly inhibited non-specific cellular uptake. Further modification of the surface to PFBD-PEG_2K_-RGD endowed the CPNs with specificity required for targeted cancer cell imaging.

PLGA is widely used for preparing polymeric NPs and is reported to benefit the NPs–cell interaction and enhance cellular uptake efficiency [81]. PCPDTBT@PEG-PLGA CPNs and PCPDTBT@PEG-DPPE CPNs exhibited higher uptake by J774A.1 cell when the suspensions were incubated in advance with mouse serum which emphasized the important role of the protein corona (PC) formation on the NP’s surface during interactions with J774A.1 cells [33]. Among these CPNs, PCPDTBT@PEG-PLGA CPNs have a higher take-up degree than PCPDTBT@PEG-DPPE CPNs. Comparing the PCPDTBT@PEG_2K_-PLGA_4K_ CPNs and PCPDTBT@PEG_5K_-PLGA_55K_ CPNs, the uptake degrees are more or less the same. The results suggest that, the choice of the copolymer type dictates the cell-particle interactions more than the chain length molecular weight of the copolymer. The authors also reported that PCPDTBT@ PEG_2K_–PLGA_2K_ accumulated more rapidly in the liver, whilst PCPDTBT@PEG_2K_-DPPE demonstrated a higher level of accumulation in tumour tissues and a longer plasma circulation half-life.

Generally, the copolymer shells present a convenient avenue to control the cell affinity, selectivity and uptake of the CPNs, with each of the copolymers presenting their own opportunities in this regard. CPNs shelled with all three groups of copolymers under consideration here have been tested in cellular cultures - reports of these tests are summarized in Table 3.

### 4.1. Cpns in Serum-Containing Environments

Once introduced into a biological system, the CPNs encounter the biological environment of systemic circulation that includes circulating proteins; these proteins are able to bind to the CPNs surface, resulting in the formation of what is referred to as the PC [86]. PC plays an essential role in making the NPs easily recognised by the innate immune system and causing quick clearance by the phagocytic cells [86]. However, to date, there has been very limited research performed in the area of interactions of PC formed on CPNs surface with phagocytic cells.

**Figure 5:**
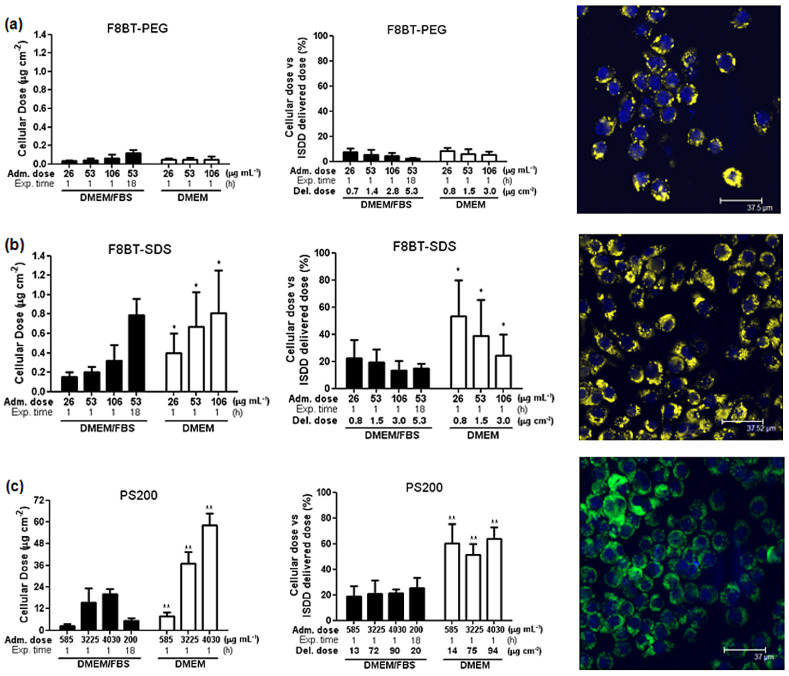
Cellular dose (left) and cellular dose as a percentage of the diffusion dosimetry model (ISDD)-delivered dose for J774A.1 cells incubated with (**a**) F8BT-PEG CPNs, (**b**) F8BT-SDS CPNs, and (**c**) PS200. Exposure conditions are described on the abscissa and include administered dose (μg.mL^−1^), exposure time (h) and the corresponding ISDD delivered dose value (μg.cm^−2^). Data represent the mean ± SD of n = 4 independent experiments (* p<0.05, ** <0.01, *** <0.001). The right panel depicts fluorescence micrographs of J774A.1 cells exposed to F8BT-PEG, F8BT-SDS and PS200 in DMEM/FBS (administered dose: 53, 53, and 200 μg.mL^−1^; incubation time: 18 h; ISDD delivered dose: 5.3, 5.3, and 20 μg.cm^−2^, respectively). Reprinted with permission from [54]. In the left and central columns of (**a**–**c**), the black and white bars correspond to data obtained for J774A.1 cells in DMEM/FBS and DMEM, respectively. Copyright 2015 American Chemical Society.

In one of a few studies published in this area, Khanbeigi et al. used CPNs of F8BT stabilized with ionic surfactants sodium dodecyl sulfate (SDS) or nonionic surfactants PEG and compared them with polystyrene NPs of a similar size (PS200) [54]. They investigated the differences in PC formation after incubation with a serum-containing medium among the three samples and studied the biological performance of phagocytic J774A.1 macrophage cells. They found that all three NPs did not aggregate in DMEM/FBS over a 24 h incubation period, whilst F8BT@SDS and PS200 NPs aggregated after about four hours in serum-free DMEM and F8BT@PEG NPs remained stable over 24 h. As shown in Figure 5, they also noticed that unlike PS200 enrichment of several serum proteins onto the particle surface, F8BT CPNs did not enrich specific proteins onto the NPs surface. F8BT@PEG CPNs showed the minimal (<5%) cell uptake, whereas that of F8BT@SDS CPNs was 20% and that of PS200 was 60% [54]. This phenomenon is consistent with previous observations that PEG encapsulation reduces NP recognition by phagocytic cells through steric repulsion effects, resulting in longer circulation times [87].

### 4.2. Effects of Zeta Potential and Size of CPNs

Typically, the zeta potential of NPs plays an essential role in influencing their interaction with cells. For example, previous studies have shown that charged polystyrene and iron oxide particles are taken up better than their lesser-charged counterparts by both phagocytic and non-phagocytic cells [88,89]. When it comes to CPNs, however, the link between the zeta potential and the cellular uptake is less clear, with no clear identifiable trends between the two. The issue is somewhat aggravated by the lack of comprehensive and quantitative analysis of cellular uptake of CPNs, as is exemplified by the work of Bourke et al. who show that both MEH-PPV@PSMA (−30mV) and MEH-PPV@F127 (−10mV) can enter HeLa cells [46] but fail to specify any quantitative differences between the two. Our own analysis of the relevant reports (Table 3) suggests that, in addition to zeta potential, the cellular uptake of CPNs by a specific cell line is influenced by the composition of the copolymer shell [69] and also the MW of the copolymer that is used as a stabilizing shell [56]. In addition, the presence and absence of relevant target ligands on CPN surface has a strong impact on the cellular interactions; as such, modification of CPNs with targeting ligands causes significant changes in their cellular uptake ability [58,65]. For example, Wang et al. observed that the strongly charged PTPEDC@DSPE-PEG-Mal CPNs (−44.5to−46.6mV) fail to enter HeLa cells, but they are easily internalized by the same cells post-modification with Tat (−2.5to−6.6mV) [48]. The authors attribute this change in the uptake behaviour to the presence of the target ligand. All of the above studies reveal a gap in our knowledge of the mechanisms by which CPNs are uptaken into cells and the effect that surface charge has on these mechanisms. Further studies are urgently needed in this area, even if to confirm findings previously observed for other types of nanoparticles. This could include, for example, observations of internalization of anionic NPs *via* chlathrin-mediated endocytosis and cavelolae-mediated endocytosis in HeLa cells, but *via* chlathrin-mediated endocytosis mechanism only for cationic NPs [90,91].

Investigations of the effect of NPs size on their cellular uptake have equally identified it as a key parameter in determining the mechanism by which the uptake occurs. It has been shown that the cellular uptake of NPs of 20–100 nm in diameter occurs mainly through caveolin-mediated endocytosis, whilst that of NPs of 100–350 nm size happens is primarily through clathrin-mediated endocytosis [92]. Generally, NPs in the size range of 15–260 nm can be taken up efficiently or stain the cellular membrane of different cell lines, whilst sizes smaller than 150 nm are preferable for applications involving live imaging in mice [10]. Since most nanoparticles of CPs fall within these size ranges (Table 1), they can be deemed to be suitable for biomedical or bioimaging applications. CPNs encapsulated in copolymer shells do offer one noteworthy advantage—their hydrodynamic sizes can be easily tunable *via* the molecular weight of the copolymer chains. For example, Hong et al. were able to tune the hydrodynamic size of pDA CPNs by coating them with DSPE-mPEG copolymers of different MW (2 kDa and 5 kDa), obtaining smaller CPNs when using shorter PEG chains [63]. Qiao et al. have also experimented with using different mass ratios of hydrophobic to hydrophilic units in their copolymer, as well as different overall MW of the copolymer, as means of controlling CPN size, showing that more hydrophobic character of the copolymer shell and its larger MW resulted in an increase of CPN sizes [93] from 40 nm to 200 nm. This work points towards a possibility of using such approaches to select specific mechanisms of cellular uptake for the developed CPNs.

In summary, above works make it abundantly clear that the copolymer shell plays a crucial role in determining the nature of interactions between the CPNs and the nearby cells, in many cases also determining their final destination, i.e., whether they are internalized into the cell, attached to cell membranes or have no affinity to the specific cell lines (see Table 3 for the summary). However, within this topic, examples in the literature are too few and often not quantitative enough to draw any concrete conclusions as to any possible trends between the three groups of copolymers that we are considering in this review.

## 5. Multimodal CPNs-Based Probes

Several CPNs-based systems, stabilized by various copolymers, have been investigated for multimodal applications, including those that incorporated photo-activated therapeutic modalities. Here, some of these reports are reviewed, with a specific emphasis on the modification of photothermal and photodynamic properties of CPNs upon the addition of copolymer as a solubilizing shell.

### 5.1. Photothermal Properties

**Figure 6:**
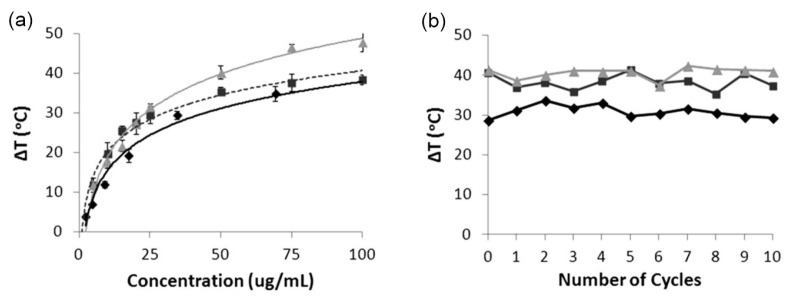
Photothermal properties of PCPDTBSe CPNs. A comparison of (**a**) the heating efficiency and (**b**) heating reproducibility curves for bare PCPDTBSe NPs (black diamond), PW-PCPDTBSe NPs (grey triangles) and PW-PCPDTBSe nanofibers (dark grey squares). In (**b**), the concentration of CPNs was 50 μg.mL^−1^. Laser parameters were 3 W and 1 min for both measurements. Error bars are shown as standard deviation of the mean. Adapted from [61] with permission from John Wiley and Sons.

A single report exists, comparing the photothermal properties of bare and copolymer-solubilized CPNs, making it impossible to draw any conclusions as to any possible dependencies of photothermal properties of CPNs on the material composition of the stabilizing shell. Interestingly, however, the same study suggests that the photothermal properties of CPs materials might also depend on their shape. As shown in Figure 6, MacNeill et al. compared the photothermal performance of PCPDTBSe@F127 CPNs, nanofibres and bare PCPDTBSe CPNs [61]. The results showed that at lower concentration (10 μg/mL), PCPDTBSe@F127 nanofibers showed the most remarkable increment in temperature. At a higher concentration (100 μg/mL), PCPDTBSe@F127 CPNs showed a much higher temperature increase ($\Delta T\approx 47{\;}^{{}^{\circ}}$C), when compared to the other two samples ($\Delta T\approx 35{\;}^{{}^{\circ}}$C). They concluded that F127-encapsulate PCPDTBSe CPNs and nanofibers outperform bare PCPDTBSe NPs in generating more significant heat that can be exploited to destroy colorectal cancer cells, but that the shape of NPs, as well as the concentration of the sample also influence the photothermal capacity of the CPNs [61]. The dependency of PTT performance on the shape of the nanoparticle does not only exist in CPNs; other types of NPs, such as plasmonic NPs, also have similar dependency, although likely owing to different photophysical phenomena [94].

It should be noted that the use of F127 may not be optimum for stabilization of CPNs intended for PTT applications. In their work on F127 micelles loaded with BBT-EHT molecular dye, Huang et al. observed some agglomeration of the F127 micelles upon their irradiation with near infrared light [95]. They attributed this to low lower critical solution temperature of the Pluronic F127, causing gel formation at relatively low temperatures achieved during the PTT application of their micelles. As a result of this observation, they opted for PEG-derived stabilizer instead of F127 in their follow-up study involving BBT-2FT molecular theranostic agent [96], reporting no such agglomeration in this case. This being said, the CP used in CPNs are distinctly different to small molecules such as BBT-EHT and BBT-2FT, and as such concerns over agglomeration of F127 in these studies may not be translatable to the CPN-based systems. Due to the lack of reports in this area, however, the suitability of F127 for CPN-based PTT probes remains to be an open question.

### 5.2. Photodynamic Properties

**Figure 7:**
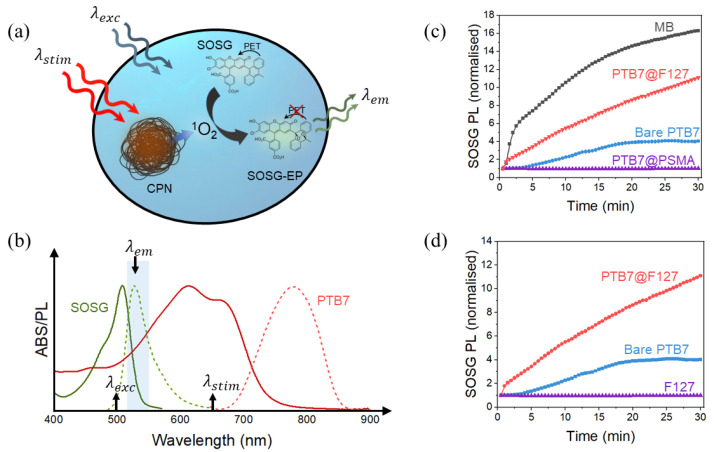
Measurements of singlet oxygen production by PTB7 CPNs using the SOSG chemical sensor. (**a**) The measurement scheme consisted of continuous stimulation of singlet oxygen production by the CPNs, converting SOSG into its fluorescent form, SOSG-EP, via a reaction with singlet oxygen, and recording SOSG-EP to evaluate the amount of singlet oxygen produced by the CPNs. The arrows in panel (**b**) indicate the relevant wavelengths used in the experiments and normalised absorption and emission spectra of SOSG and PTB7 CPNs. Panel (**c**) shows the temporal evolution of the SOSG-EP fluorescence signal for different types of CPNs and a reference photosensitiser (methylene blue, MB). Panel (**d**) compares singlet oxygen production by the PTB7@F127 CPNs, the bare PTB7 CPNs, and the F127 copolymer. For all measurements, samples had absorbances of ≈0.5 at the stimulation wavelength (635 nm) before adding SOSG. This corresponded to CPNs samples’ concentrations of ≈10 μg.mL^−1^ and a concentration of 7.6 μM for the MB solution. Reprinted with permission from [69]. Copyright 2021 American Chemical Society.

According to the reports reviewed as part of this literature review, there is no reported usage of PSMA-solubilized CPNs for multimodal applications involving PDT (Table 4). This is consistent with own research showing that PSMA can quench the production of singlet oxygen production from the PTB7 CPNs, as can be seen in Figure 7 below [69]. This conclusion, however, is yet to be confirmed for other CPNs that are composed from CPs capable of photosensitization of singlet oxygen. That being said, the use of PSMA in CPN-based probes for dual imaging/PDT applications may still be possible, but most likely it will have to rely generation of ROS other than singlet oxygen. For example, in the above-mentioned work, PTB7@PSMA CPNs were shown to generate superoxide anion under UV illumination.

The results presented in Figure 7 also speak to the potential of F127 as a solubilizing copolymer of CPNs intendent for multimodal applications involving PDT. As can be seen from panel (c) of the figure, the addition of the F127 shell not only did not quench the production of singlet oxygen by PTB7 CPs, but in fact improved it by 7.5 times (when integrated over 2 h period). The ability of F127-shelled CPNs to successfully produce singlet oxygen has also been reported by Wang et al. for PBTB-based CPNs [70] (Table 4). Since singlet oxygen is produced via a triplet-triplet energy transfer between the photosensitizer and ground state molecular oxygen, it follows that F127 promotes such photophysical processes. Further reports also show that F127 can boost photoinduced electron transfer (PET) processes, leading to improved sensitivity of PET-based chemical sensors [57].

Of the three copolymer groups under review here, PEG-based copolymers are the most widely used class of stabilizing copolymers for biological applications, which include a combination of bioimaging, PTT and/or PDT capabilities (Table 4). For example, Zhang et al. fabricated multifunctional lipid-micelles comprised from PCPDTBT and Ce6 molecules within the core of the CPN, and a lipid–PEG shell conjugated with gadolinium-1,4,7,10-tetraacetic acid [97]. The prepared CPNs had excellent MR- and PA-imaging contrast-enhancement ability and simultaneously combined PTT and PDT. Another popular option for development of multi-modal probes based on CPNs is DSPE-PEG, which has been widely utilized as a stabilizing shell for probes designed for imaging-guided PTT, where the photosensitizing ability of the CPs needed for PDT application was supplemented by their photoluminescence or photoacoustic properties, enabling the imaging modality of the probes [51,73,83].

Developing multifunctional cellular probes with high selectivity has great significance in biological applications. PEG-based amphiphilic copolymers with different end groups that can be modified have been widely used to achieve targeted applications. For example, Pu et al. utilized copolymer N_3_-PEG-NH_2_, in which the -N_3_ end is conjugated to conjugated polymer PFVBT while the -NH_2_ end is modified with folic acid to develop cellular probe [29]. The same group also utilized the -NH_2_ end connected to doxorubicin to achieve PDT and chemo-therapy at the same time [72]. PEG copolymers have also been used in donor-acceptor-type CPNs, resulting in CPNs with excellent resistance to photobleaching when applied to PDT and PTT simultaneously [60].

Unfortunately, very limited number of reports exist on the use of CPNs for PDT or PTT applications, and within those only our work compares the performance of CPNs solubilized with different copolymers or with non-shelled CPNs. As such, no conclusive evidence exists for the advantageous use of one specific copolymer group when designing multi-modal probes. Nonetheless, we hope that examination of existing works on application of CPNs as photosensitizers, summarized in Table 4, can provide some guidance in the design of multi-modal CPN-based probes that incorporate PDT as one of the modalities.

## 6. Biocompatibility and Cytotoxicity

Cellular uptake, body distribution, and clearance are all affected by CPNs’ size and surface properties [39]. Previous works show that the type of surfactants used for CPNs preparation plays an essential role in their biocompatibility and in PC formation on their surfaces [38]. After administration, the surface of NPs can interact with biomacromolecules in the physiological environment and form PC by adsorption of these macromolecules [98]. More specifically, the protein adsorption is dependent on the size, composition and charge of the NPs surface. Compared to the cationic and anionic systems, NPs with a neutral surface charge tend to show more negligible protein adsorption and lower non-specific cellular uptake and an increased circulation time [99,100].

Although positively charged particles are generally taken up better than negatively charged ones, cationic NPs tend to have more significant cytotoxicity as cationic NPs appear to cause greater plasma-membrane disruption [89]. As we have seen previously, the choice of stabilizing copolymer is an important variable in determining the zeta-potential of the CPN (see Table 1; as such, it provides a viable route to optimise the electric state of the CPNs’ surfaces for specific applications. Liu et al. followed this approach by continuously varying the zeta-potential of poly[9,9-bis(2-ethylhexyl)fluorene]@PEI-PCL-PEG CPNs from −40 mV to +30 mV via *(*via) coating of their CPNs with a cationic folate-conjugate. They observed that cytotoxicity of the CPNs increased in line with zeta-potential increases, which they ascribed to increased disruption within cells [49]. This report highlights the careful interplay between many different parameters that determine the suitability of the probe for a specific application, which can be influenced by the choice of the copolymer and its subsequent functionalization: the functionalization of the probe with a targeting element endows it with a specificity that is required for bioimaging applications; however the same process results in an increased cytotoxicity of the probe.

**Figure 8:**
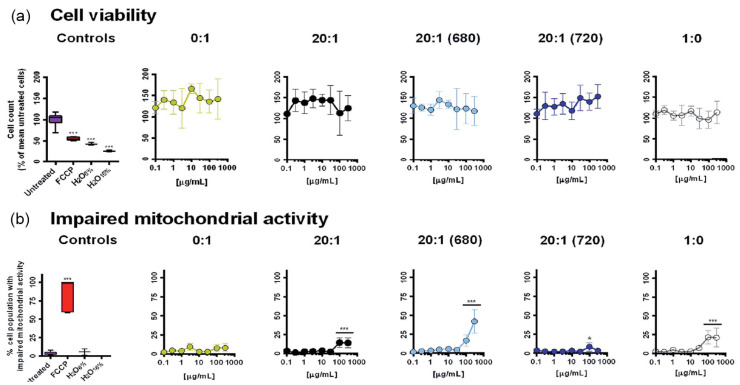
Biocompatibility of CN-PPV@PLGA-PEG CPNs. (**a**) THP-1 cell count per well after incubation for 24 h with various treatments. (**b**) Percentage THP-1 cell population with impaired mitochondrial activity (defined as a MitoTracker Red fluorescence intensity < 2000 a.u.). Values represent the mean ± standard deviation from *n* = 3 experiments with different THP-1 passage numbers (* p<0.05, ** p<0.01, *** p<0.001). Adapted from reference [37] with permission from the Royal Society of Chemistry.

Abelha et al. found that, coating CN-PPV or F8BT CPs with PEG_5K_–PLGA_55K_ copolymer did not cause any changes in the size and optical properties of the CPs; however the addition of the copolymer did provide a neutral net electrical charge to the NPs surface, which is beneficial for biomedical applications [38]. In a related work, and as shown in Figure 8, CN-PPV encapsulated with PLGA-PEG with different PLGA-to-PEG mass ratios and encapsulated small amounts (0.5–0.8 % *w/w*) of small molecular near-infrared dyes, NIR680 and NIR720, showed excellent biocompatibility when used on HeLa cell cultures, although it did seem to increase the percentage of cell population with impaired mitochondrial activity [37].

In search for a copolymer that minimizes cytotoxicity, F127 seems to have a unique advantage in providing CPNs with good biocompatibility. Bourke et al. synthesized MEH-PPV encapsulated by F127 and PSMA, and found that F127 had lower cytotoxicity compared to those encapsulated by PSMA [46]. F127-shelled PCPDTBSe CPNs and nanofibres produced by MacNeill et al. showed significantly different physicochemical properties, but all appeared to have no significant dark toxicity towards either CT26 cancer cells or FHs74 noncancerous cells, confirming their good biocompatibility [61]. In line with previous discussions of further modification being an important factor in determining CPNs biocompatibility, further doping of F127 can equally affect the biocompatibility of the CPNs. For example, in their work Bourke et al. observed tolerated cytotoxicity in HEK cells caused by low concentrations of CN-PPV CPNs encapsulated with silica-shell cross-linked F127, with the toxicity increasing to lethal at high probe concentration [45].

## 7. Conclusions and Outlook

This review considered the effects of different solubilizing copolymers on the physical and chemical properties and biological applications of CPNs. In general, the literature confirms that CPNs show minimal toxicities that can be tuned via appropriate selection of solubilizing copolymer and their further functionalization; as such, they present prominent advantages over other NPs in biological applications. However, it is also clear that copolymer selection is crucial in determining many of the physicochemical properties of CPNs, including their water solubility, size, surface potential, photo- and colloidal stability, optical properties, cellular endocytosis, cell targeting and cell imaging, PC formation and blood circulation time, changes in photodynamic and photothermal properties and the above mentioned biocompatibility. The interrelations between these parameters are complex and sometimes coupled in opposing or complementary trends. When combined with the limited literature exploring these exact interrelations and how they are affected by a range of different copolymers, it becomes difficult to draw concrete conclusions. Some general themes do emerge, however.

The use of F127 copolymer offers generally excellent biocompatibility and an ability to enhance charge and energy transfer processes, such as those that may be required for PDT applications. Its poor unspecific cellular affinity make it impractical for general imaging applications, but equally, it provides opportunities for development of target-specific probes. On the other hand, there are some concerns over its use in the development of CPN-based PTT probes due to its low lower critical solution temperature, that could lead to agglomeration of CPNs in these applications.

PSMA, on the other hand, has (unspecific) affinity to many different cell types and easily accessible carboxyl groups available for further functionalization, if specificity needs to be achieved. Furthermore, it quenches photo-induced generation of (at least) singlet oxygen by the CPs and so it can render such a CPs non-toxic. This would suggested it to be the most suitable choice as a stabilizing copolymer when only the imaging modality is desired.

For drug delivery and in vivo biological applications, PEG is most suitable shell material because it can inhibit the fast recognition of foreign bodies by the immune system, leading to a longer blood circulation time. Like F127, it does not quench ROS production by the CPNs, and so is also a good candidate for PDT-based applications, although it is not know whether its presence enhances any photophysical processes.

In summary, conjugated polymer NPs solubilized with copolymers present a promising and an exciting class of materials for development of theranostic probes. Amongst the many beneficial properties, however, lie many gaps in our knowledge and in our understanding of the different interrelations between CPNs composition and their physicochemical and biocompatibility properties. It is our hope that, in identifying these gaps, we enable the research in this highly promising field so that it may reach its full potential.

## Figures and Tables

**Table 2:** Characteristics of the CP-loaded PLGA NPs [58].

	PF	PFV ^1^	PFBT	MEH-PPV
Particle size ^2^ (nm)	261.2±4.5	257.5±4.2	242.9±3.8	271.4±5.2
Polydispersity	0.129±0.034	0.124±0.031	0.120±0.028	0.158±0.037
Zeta potential (mV)	−36.61	−37.15	−33.42	−35.25
Encapsulation efficiency (%)	≈41.3	≈43.9	≈44.2	≈47.6
Leakage of CPs in 5 days (%)	0.095	0.098	0.087	0.062

^1^ For pure PFV NPs prepared without PLGA the particle size was 221±7.3 nm, and the polydispersity was 0.186±0.049. ^2^ Determined by dynamic light scattering (DLS) measurements.

**Table 3:** Summary of in vitro applications of CPNs.

Cell Line	Core Material	Shell Material (Target Ligand)	Application *	Uptake ^#^	Refs.
MCF-7	PFPtTFPP	PSMA	IMG	unclear	[43]
	P1-P4	PSMA (anti-EpCAM)	IMG	membrane	[82]
	PFVBT + PIDTTTQ	DSPE-PEG_2K_-Mal (anti-HER2)	IMG	no	[71]
	PFVBT	N_3_-PEG-NH_2_ (cRGD)	CT	-	[72]
	CPE	N_3_-PEG-NH_2_ (FA)	IMG	uptake	[29]
	HCPE	N_3_-PEG-NH_2_	IMG	yes	[56]
	PFBD-N_3_	NH_2_-PEG-COOH (RGD)	IMG	no	[65]
	F8BT	PS-PEG-COOH (IgG)	IMG	membrane	[30]
	PFBT	PSMA (click reaction)	IMG	membrane	[20]
	PF/PFV/PFBT/MEH-PPV	PLGA (FA)	IMG	yes	[58]
U87 Glioma	P1 (BDT + BBT)	DSPE-PEG_2K_	PA	unclear	[83]
	pDA	DSPE-mPEG (erbitux)	IMG	no	[63]
J774A.1	F8BT	PEG	CT	-	[54]
	PCPDTBT	PEG-PLGA	CT	-	[38]
KB	Poly[9,9-bis(2-ethylhexyl)fluorene]	PEI-PCL-PEG (FA)	IMG / CT	unclear	[49]
A549	PFP/PFQ/F8BT/PFBO	PS-PEG-COOH	CT	-	[55]
	PTB7	PSMA	IMG	unclear	[69]
SK-BR-3	P1-P4	PSMA (anti-EpCAM)	IMG	membrane	[82]
	PFVBT + PIDTTTQ	DSPE-PEG_2K_-Mal (anti-HER2)	IMG	yes	[71]
	F8BT	PS-PEG-COOH (IgG)	IMG	membrane	[30]
NIH 3T3	SP2	PEG-b-PPG-b-PEG	CT	-	[84]
	PFVBT + PIDTTTQ	DSPE-PEG_2K_-Mal (anti-HER2)	IMG	no	[71]
	CPE	N_3_-PEG-NH_2_ (FA)	IMG	no	[29]
	HPCE	N_3_-PEG-NH_2_	CT	-	[56]
	DPP-TT	DSPE-mPEG_5K_	CT	-	[51]
	PF/PFV/PFBT/MEH-PPV	PLGA (FA)	IMG	no	[58]
	BPSB unit (S2 and M2)	PSMA	IMG	membrane	[76]
4T1	CP	PSMA	CT	-	[50]
	BT-BIBDF	PEG-PCL	CT	-	[60]
	CP1-CP4	DSPE-mPEG_2K_	CT	-	[85]
MDA-MB-468	pDA	DSPE-mPEG (erbitux)	IMG	membrane	[63]
MDA-MB-231	PBIBDF-BT	mPEG-b-PHEP	IMG	uptake	[59]
	PFVBT	N_3_-PEG-NH_2_	IMG	yes	[72]
HeLa	PTPEDC	DSPE-PEG-Mal (Tat)	IMG	unclear	[48]
	PDPP-DBT	DSPE-PEG-Mal (Tat)	CT	-	[47]
	P1-P4	PSMA (anti-EpCAM)	IMG	no	[82]
	PCPDTBT + PC70BM	PEG-b-PPG-b-PEG	CT	-	[62]
	PBTB	F127	CT	-	[44]
	MEH-PPV	F127	IMG/CT	unclear	[46]
	MEH-PPV	PSMA	IMG/CT	unclear	[46]
	CN-PPV	F127 + TMOS	IMG	yes	[45]
	CN-PPV	PEG-PLGA	IMG	unclear	[37]
	PBMC	PSMA	CT	-	[15]
	DPSB unit (S2 and M2)	PSMA	IMG	membrane	[76]
	DPP-TT	DSPE-mPEG_5K_	IMG	unclear	[51]
	PFTBT5 + PFO	PSMA	CT	-	[68]
BS-C-1	PFTBT5 + PFO	PSMA	IMG	yes	[68]
HepG-2	PtTFPP + PFO	poly-L-lysine	IMG/CT	yes	[70]
	DPSB unit (S2 and M2)	PSMA	IMG/CT	membrane	[76]
HT 29	PFBD-N_3_	NH_2_-PEG-COOH (RGD)	IMG/CT	yes	[65]
HCE	MEH-PPV	PSMA	IMG/CT	unclear	[46]
	MEH-PPV	F127	IMG/CT	unclear	[46]
HEK 293	MEH-PPV	PSMA	CT	-	[46]
	CN-PPV	F127 + TMOS	CT	-	[45]
FHs 74 Int.	PCPDTBSe	F127	CT	-	[61]
CT-26	PCPDTBSe	F127	IMG/CT	unclear	[61]
WPE1-NB26	CN-PPV	F127 + TMOS	IMG	unclear	[45]
WPE1-NA22	CN-PPV	F127 + TMOS	IMG	unclear	[45]
RWPE-1	CN-PPV	F127 + TMOS	IMG	unclear	[45]

* IMG = confocal imaging; CT = cytotoxity investigation; PA = photoacoustic imaging; # Here “membrane” refers to CPNs adhering to the cells; “no” refers to no uptake of the cells and no adhesion to them; “unclear” indicates that data provided was not sufficient to make a conclusion; “-” indicates non-reporting of the uptake data; “yes” denotes uptake and confirmed internalization of CPNs by the cells.

**Table 4:** CPNs as photosensitizers.

Core Material	Shell Material	ROS Detected ^†^	Assay/Sensor Used ^#^	Irradiation Conditions *	Refs.
PTB7	F127	singlet oxygen	SOSG	635 nm CW, 4.5 mW	[69]
		superoxide anion	chronoamperometry	UV lamp	[69]
		intracellular ROS	DCFDA assay	660–850 nm, 10 J.cm^−2^	[69]
	PSMA	*no*	SOSG	635 nm CW, 4.5 mW	[69]
		superoxide anion	chronoamperometry	UV lamp	[69]
PTPEDC	DSPE-PEG-Mal	singlet oxygen	ABDA,DCFDA	400–700 nm, 50 mW.cm^−2^	[48]
PFVBT	DSPE-PEG${}_{2K}$-Mal	singlet oxygen	DCFH,ABDA	60 s CW WL at 0.25 W.cm^−2^	[71]
			DCFDA	30 s CW WL at 0.25 W.cm^−2^	[71]
BT-BIBDF	PEG-PCL	singlet oxygen	DPBF, ESR	60 s CW WL at 0.25 W.cm^−2^	[65]
			DCFDA	30 s CW WL at 0.25 W.cm^−2^	[65]
PBTB	F127	singlet oxygen	RNO	254 nm, 2 W.cm^−2^	[44]
			DCFDA		[44]
PtTFPP + PFO	poly-L-lysine	singlet oxygen	ADMA, DPBF	540 nm	[70]
			MTT	405 nm, 0.03 and 0.06 W.cm^−2^; 740 nm, 3.0 W.cm^−2^	[70]
CP1-CP4	DSPE-mPEG${}_{2K}$	singlet oxygen	ABDA	400–700 nm, 60 mW.cm^−2^	[85]
			DCFDA, PI, MTT		[85]

* CW = continuous excitation; WL = white light. ^#^ Please see the abbreviations section for the definitions used here. ^†^ If ROS species were detected, then the entry describes the type of ROS detected. “*no*” indicates a negative result for the test, i.e., that the ROS tested for was not produced by the CPN.

## Abbreviations

The following abbreviations are used in this manuscript:

ABDA	9,10-Anthracenediyl-bis(methylene)dimalonic acid
ADMA	Assymetric dimethylarginine
BDT	Benzodithiophene
BBT	Benzobisthiadiazole
BT	Bithiophene
BIBDF	(3E,7E)-3,7-Bis(2-oxoindolin-3-ylidene)benzo-[1,2-b:4,5-b${}^{\prime }$]-difuran-2,6(3H,7H)-dione
BIBDF	Bis(2- oxoindolin-3-ylidene)-benzodifuran-dione
BBT-EHT	Benzo[1,2-c;4,5-c0]bis[1,2,5]thiadiazole-4,7-bis(5-(2-ethylhexyl)thiophene)
BBT-2FT	Benzo[1,2-c;4,5-c0]bis[1,2,5]thiadiazole-4,7bis(9,9-dioctyl-9H-fluoren-2-yl)thiophene
CP(s)	Conjugated Polymer(s)
CPE	Conjugated polyelectrolyte
CPN(s)	Conjugated Polymer Nanoparticle(s)
CT	Cytotoxicity investigation
CN-PPV	Poly(2,5-di(hexyloxy)cyanoterephthalylidene)
DBT	1,4-dithienylbenzothiadiazole
DCFDA	2${}^{\prime }$,7${}^{\prime }$-Dichlorodihydrofluorescein diacetate
DPBF	1,3-Diphenylisobenzofuran
DLS	Dynamic light scattering
DMEM	Dulbecco modified essential medium
EPR	Enhanced permeability and retention
ESR	Electron spin resonance
FA	Folic acid
F8BT	Poly(9,9-dioctylfluorene-2,1,3-benzothiadiazole)
FDA	Food and drug administration
F127	Pluronic poly(ethylene glycol)-*block*-poly(propylene glycol)-*block*-
	-poly(ethylene glycol) diacrylate
IMG	Confocal imaging
NP(s)	Nanoparticle(s)
Mal	Maleimide
MEH-PPV	Poly[2-methoxy-5-(2-ethylhexyloxy)-1,4-phenylenevinylene]
MR	Magnetic resonance
MTT	3-(4,5-Dimethylthiazol-2-yl)-2,5-diphenyltetrazolium bromide
MW	Molecular weight
HCPE	Hyperbranched conjugated polyelectrolyte
HER2	Human epidermal growth factor receptor 2
P1	Poly[9,9-bis(6${}^{\prime }$ -(N, N -dimethylamino)hexyl))fluorenyldivinylene-alt-4,7-
	(2${}^{\prime }$,1${}^{\prime }$,3${}^{\prime }$,-benzothiadiazole) dibro-mide]
P2	poly[9,9-bis(N-(but-3${}^{\prime }$-ynyl)-N,N-dimethylamino)hexyl))fluorenyldivinylene-alt-
	4,7-(2${}^{\prime }$,1${}^{\prime }$,3${}^{\prime }$,-benzothiadiazole) dibromide]
PA	Photoacoustic imaging
PBMC	poly[3-2-[2,5-Bis-(2-ethyl-hexyloxy)-4-propenyl-phenyl]-vinyl-9-butyl-6-methyl-
	9H-carbazole]
PBS	Phosphate-buffered saline
PC	Protein corona
PC70BM	(6,6)-phenyl-C71-butyric acid methyl ester
PCBM	(6,6)-phenyl-C61-butyric acid methyl ester
PCPDTBT	Poly[2,6-(4,4-bis(2-ethylhexyl)-4H-cyclopenta- [2,1-b;3,4-b${}^{\prime }$]dithiophene)-alt-
	4,7-(2,1,3-benzothiadiazole)]
PDA	Poly(benzo[1,2-b:3,4-b${}^{\prime }$]difuran-alt-fluorothieno-[3,4-b]thiophene)
PCPDTBSe	poly[4,4-bis(2-ethylhexyl)-cyclo-penta[2,1-b;3,4-b${}^{\prime }$]dithiophene-2,6-diyl-alt22,1,3-
	benzoselena-diazole-4,7-diyl]
PDHF	Poly(9,9-dihexylfluorene)
PDPP3T	Poly((2,5-diyl-2,3,5,6- tetrahydro-3,6-dioxo-pyrrolo(3,4-c)pyrrole-1,4-diyl)-
	alt-(2,2${}^{\prime }$:5${}^{\prime }$,2${}^{\prime \prime }$-terthiophene-5,5${}^{\prime \prime }$-diyl))
PDPP-DBT	Poly[2,6${}^{\prime }$-4,8-di(5-ethylhexylthienyl)benzo[1,2-b;3,4-b]dithiophene-alt-5-
	dibutyloctyl-3,6-bis(5-bromothiophen-2-yl)pyrrolo[3,4-c]pyrrole-1,4-dione]
PDT	Photodynamic therapy
PEG	Polyethylene glycol
PET	Photoinduced electron transfer
PEO	Poly(oxyethylene)
PF	Poly[9,9-bis(2-ethylhexyl)fluorene]
PF-2	Poly[9,9-dihexylfluorene-alt-9,9-bis(2-(2-(2-methoxyethoxy)ethoxy)ethyl)fluorine]
PFBD-N${}_{3}$	Poly(9,9-bis(6${}^{\prime }$-azidohexyl)fluorene-alt-2,1,3-benzoxadiazole)
PFBO	2,1,3-Benzooxadiazole-alt-fluorene
PFBT	Poly[9,9-bis(2-(2-(2-methox-yethoxy)ethoxy)ethyl)fluorene-alt-4,7-
	(2,1,3-benzothiadiazol)]
PFO	Poly(9,9-dioctylfluorenyl-2,7-diyl)
PFODBT	Poly[2,7-(9,9-dioctylfluorene)-alt-4,7-bis(thiophen-2-yl)benzothiadiazole]
PFPE	Poly(2,7-(9,9-hexylfluorene)-alt-4,4${}^{\prime }$-phenylether)
PFPtTFPP	Poly((2,7-dibromo-9,9-dioctyl-9H-fluorene)(9,9-dioctyl-9H-fluorene-2,7-diboronic acid
	bis(pinacol)ester)(platinum(II) 5,15-bis(pentafluorophenyl)-10,20-bis(4-bromophenyl)
	porphyrin))
PFPV	Poly[9,9-dioctyl-2,7-divinylene-fluorenylene-alt-co-(2-methoxy-5-(2- ethylhexyloxy)-
	1,4-phenylene)]
PFTBT5	Poly(9,9-dioctylfluorene)-co-(4,7-di-2-thienyl-2,1,3-benzothiadiazole)
PFV	poly[9,9-bis(2-(2-(2-methoxyethoxy)ethoxy)ethyl)fluorenyldivinylene-alt-9,9-bis
	(3-t-butylpropanoate)fluorene]
PFVBT	Poly[9,9-bis(N-but-3${}^{\prime }$-ynyl-N,N-dimethylaminohexyl)fluorenyldivinylene-alt-
	4,7-(2${}^{\prime }$,1${}^{\prime }$,3${}^{\prime }$,-benzothiadiazole) dibromide]
PI	Propidium iodide
PIDTTTQ	Poly[(4,4,9,9-tetrakis(4-(octyloxy)phenyl)-4,9-dihydro-s-indacenol-dithiophene-2,7-
	diyl)-alt-co-4,9-bis(thiophen-2-yl)-6,7-bis(4-(hexyloxy)phenyl)- thiadiazolo-
	quinoxaline]
PLGA	Poly(D, L-lactide-co-glycolide)
PLQY	Photoluminescence quantum yield
PPO	Poly(oxypropylene)
PSMA	Poly (styrene-co-maleic anhydride)
PTB7	Poly(4,8-bis[(2-ethylhexyl)oxy]benzo[1,2-b:4,5-b${}^{\prime }$]dithiophene-2,6-diyl(3-fluoro-2-
	[(2-ethylhexyl)carbonyl]thieno[3,4-b]thiophenediyl))
PTO	polythiophene
PTT	Photothermal therapy
PtTFPP	Platinum(II)-5,10,15,20-tetrakis-(2,3,4,5,6-pentafluorophenyl)-porphyrin
QY	Quantum yield
RNO	p-Nitrosodimethylaniline
ROS	Reactive oxygen species
SDS	Sodium dodecyl sulfate
SOSG	Singlet oxygen sensor green
SP1	Poly(diketopyrrolopyrrole-altthiophene)
SP2	Poly(diketopyrrolopyrrole-altthiadiazoloquinoxaline)
Tat	TAT peptide (GRKKRRQRRRPQ)
THF	Tetrahydrofuran
